# Effectiveness of the Apple Watch as a mental health tracker

**DOI:** 10.7189/jogh.14.03010

**Published:** 2024-02-09

**Authors:** Mariam Adil, Isha Atiq, Sumaiya Younus

**Affiliations:** 1 Department of Internal Medicine, Dow University of Health Sciences, Karachi, Pakistan; 2 Institute of Professional Psychology, Bahria University, Karachi, Pakistan

In recent times, the prevalence of mental health conditions such as anxiety, depression, and posttraumatic stress disorder (PTSD) has been increasing drastically, consequently increasing suicide rates, loss in economic productivity, and substance abuse worldwide [1]. This significant rise in mental health disturbances may be attributed to deteriorating health conditions and the emergence of viral outbreaks such as the coronavirus disease 2019 (COVID-19) pandemic and the monkeypox epidemic. These aforementioned environmental conditions have had a substantial negative influence on people's mental health and daily activities. Consequently, there has been an increased demand for mental health services and a growing interest in methods to assist individuals in managing their mental well-being, including through the use of wearable technology such as smartwatches [2]. While smartwatches cannot directly quantify mental health, studies have shown that smart devices such as the Apple Watch are reliable in monitoring certain physiological indicators that can, in turn, provide indirect insights into mental health [3].

Mental health has many aspects, but mainly refers to an individual's emotional, psychological, and social well-being [4]. These factors of mental health can be assessed via the monitoring of certain physical health indicators such as heart rate (HR), respiratory rate, and sleep duration. Recently, research has been conducted to examine the potential use of the Apple Watch for monitoring mental health. Lui et al. [5] effectively determined its effectiveness as a proficient wearable device for measuring overall health and well-being by monitoring various physiological markers. These physiological markers included heart rate variability (HRV), HR, energy expenditure (EE), and sleep cycle. According to the Diagnostic and Statistical Manual of Mental Disorders (DSM) criteria, variations in sleep cycle are strongly indicative of depressive disorders. Thus, accurate monitoring of people’s sleep cycles may help specialists identify these disorders without delay [6]. Lui et al. [5] also assessed the surveillance of HRV, which indicates significant changes in physical and emotional states. However, they found that some physical factors determined using the Apple Watch, such as EE and activity expenditure, are overestimated and thus cannot be considered reliable for follow-up. Similarly, Falter et al. [7] observed that, although the Apple Watch accurately measures HR, it overestimates EE. Moreover, a comparative analysis with the Polar A370 fitness tracker suggested that both devices are inaccurate for measuring EE [8].

One possible reason for HRV being a key indicator of mental well-being is its relation to neurological function. Increased HRV levels signify a positive balance between the body's sympathetic and parasympathetic nervous systems, with an increase in sympathetic activity being associated with stress and anxiety. Abnormal HRV has been recorded in various mental disorders such as major depressive disorder, schizophrenia, and PTSD [9]. In 2018, Hernando et al. [10] compared HRV measures derived from the Apple Watch RR interval series to those obtained using a Polar H7 band as a reference; they found that HRV metrics from the former device effectively reflected changes caused by mild mental stress. Furthermore, Dooley et al. [11] found the Apple Watch to be more reliable than devices such as Fitbit and Forerunner for recording EE and HR, while Nelson et al. [12] recorded an error margin of less than 10% when measuring HR and HRV. Similarly, Thomson et al. [13] observed that the Apple Watch displayed lower relative error rates in comparison to Fitbit for the measurement of HR across a range of exercise intensity levels when using ECG as a control. Conversely, another study suggested that both Fitbit and the Apple Watch, at times inaccurately measured HR and EE [14].

Regardless, the use of the Apple Watch is not limited to its diagnostic capability; it can also be used to maintain mental well-being through the use of mindfulness and meditation applications which allow users to perform ‘mindfulness’ sessions comprising deep breathing and reflection. Studies have shown that these sessions can reduce anxiety related symptoms and stress by nearly 50%, while mindful meditation also significantly improves sleep quality, which may aid in mood regulation [15,16]. For example, the ‘Health’ application allows people to record their daily moods and have constant access to depression and anxiety tests that are used in clinical settings. These test results can then help users determine their risk levels for certain conditions and help them connect to resources available in their vicinity or generate a PDF of their results that they can share with a doctor [17]. While there are ways to assess mental health such as psychometric scales, therapy visits, and self-check lists, many individuals hesitate to use them due to financial constraints, fear of social abandonment, or denial [18]. The Apple Watch thus provides an easily accessible and widely acceptable method for people to monitor mental health and maintain mental well-being [19].

Nevertheless, the device cannot be used for the direct diagnosis and assessment of mental health conditions, given their complex nature; this process require the expertise of medical professionals, such as psychiatrists or psychologists, who apply multiple diagnostic tests to skillfully evaluate a person’s mental state. Furthermore, certain psychological conditions may require treatment with therapy or medication that only a qualified medical practitioner is equipped to provide, rather than with mental health applications. Consequently, wearables like the Apple Watch can play a supportive role in monitoring mental health and overall well-being, but should not be viewed as replacements for professional medical advice, diagnosis, or treatment. While wearable technology can complement the efforts of medical professionals in monitoring and improving mental health, it cannot substitute professional health care services [20].

It should also be noted that the accuracy of certain diameters such as HR and EE obtained during monitoring can vary during different levels of physical activity intensity. Additionally, the Apple watch may at times overestimate sleep duration and misclassify the sleep stages [12]. Moreover, the correlation between mental and physical health is complex, and individuals' responses may differ according to environmental and genetic factors. A recent study found a mere 54% accuracy in associating neural and physiological markers with mental states, thus supporting the hypothesis that mental health conditions are multifaceted, and that not all aspects can be effectively quantified through physiological indicators [21]. It can be challenging to identify if a physical symptom is related to a mental issue or an underlying physiological issue. For instance, an increase in HR may either be attributed to panic attacks, characterised by abrupt surges of intense discomfort and involuntary arousal [22], or a physiological instability, such as a metabolic disturbance or raised blood pressure [23]. Similarly, HRV is influenced by various factors, making it an unreliable indicator of mental health without further investigation. For example, Licht et al. [24] concluded that, although they noted a strong correlation between depression and reduced HRV, these reductions were actually attributable to the use of antidepressants. Thus, individuals cannot solely rely on the Apple Watch for monitoring mental health and should always consult a specialist as well. Researchers focussing on this topic should strive to conduct more studies on the use of the Apple Watch for monitoring mental health conditions.
